# Systematic review and meta-analysis of the impact of loss of consciousness on clinical outcomes in mild traumatic brain injury

**DOI:** 10.1038/s41598-025-13979-0

**Published:** 2025-08-12

**Authors:** Jasmine Omair, Victoria Alkin, Vaitheesh Jaganathan, Martin F. Bjurström, Doniel Drazin, Emily Sieg, Robert P. Friedland, Maxwell Boakye, Nicholas Dietz

**Affiliations:** 1 University of Louisville School of Medicine, Louisville, KY USA; 2 Department of Neurosurgery, University of Louisville, Louisville, KY USA; 3 Department of Surgical Sciences, Clinical Pain Research, Uppsala University, Uppsala, Sweden; 4 Department of Neurosurgery, Providence Regional Medical Center Everett, Everett, WA USA; 5 Department of Neurology, University of Louisville, Louisville, KY USA

**Keywords:** Concussion, Traumatic brain injury, Loss of consciousness, Clinical outcomes, Brain injuries, Outcomes research

## Abstract

While loss of consciousness (LOC) is a key factor in assessing head injuries, its impact on clinical outcomes, including persistent post-concussive symptoms, mental health disorders, quality of life, and neurodegeneration, remains unclear. This systematic review explores the association of LOC in Mild Traumatic Brain Injury (mTBI) with clinical outcomes such as mental health, quality of life, and risk of neurodegenerative diseases. Comprehensive systematic review methodology; two electronic databases (PubMed, Embase) were systematically searched from January 1990 to December 2024. Pooled odds ratios (OR) were obtained using a random effects model. A total of 595 studies were assessed with 30 trials meeting inclusion criteria. The presence of LOC is associated with worsened clinical outcomes including persistent post-concussive symptoms (OR 1.89, 95% CI: 1.59–2.25), post-traumatic stress disorder (OR 1.81, 95% CI: 1.54–2.12), depression (OR 2.69, 95% CI: 2.10–3.43), and overall health-related quality of life (OR 1.84, 95% CI: 1.49–2.26). These findings suggest that the role of LOC in the outcomes of mTBI supports a higher risk of poorer short and long-term outcomes. Future studies may investigate variation in post-mTBI sequelae among those with similar LOC timelines.

**Supplementary Information:**

The online version contains supplementary material available at 10.1038/s41598-025-13979-0.

## Introduction

The annual incidence of traumatic brain injuries (TBI) in the United States exceeds four million, of which 90% are classified as mild traumatic brain injuries (mTBI)^1^. Significant economic and functional impacts underlie mTBI, as nearly 20% are unable to return to work within the first year of injury^2,3^ and up to 40% develop symptoms lasting three months or longer^4,5^. The terms ‘mTBI’ and ‘concussion’ are used interchangeably throughout this manuscript, consistent with the American Congress of Rehabilitation Medicine (2023)^6^. Post-concussive symptoms now referred to as persisting symptoms after concussion (PSaC)^7^ include headaches, dizziness, and fatigue, and cognitive difficulties vary widely and arise from multiple factors, including preexisting conditions, psychological influences, and secondary medical issues^7^. Further, individuals with a history of concussion are at an increased risk for negative health outcomes as they age, including increased risk of post-traumatic stress disorder (PTSD) and dementia, with reduced health-related quality of life^8^.

Despite most TBI being categorized as mild, little is known regarding the effect of LOC with mTBI on short and long-term clinical outcomes. Mild TBI with LOC may indicate a more insidious form of brain injury than mTBI without LOC, potentially contributing to a higher risk of persistent symptoms and prolonged recovery^9^. Indeed, the mechanisms of LOC are debated, its origin is believed to be brain dysfunction involving both cortical and subcortical structures, including the brainstem and reticular activating system^10^.

This systematic review aims to explore the role of LOC in mTBI and how it affects short and long-term clinical outcomes. Given its potential impact on injury severity and negative effects on prognosis, the presence of LOC in mTBI may influence clinical practice and guidelines regarding return to activity.

## Methods

For this systematic review, mTBI was defined in accordance with the American Congress of Rehabilitation Medicine criteria as a condition caused by a plausible biomechanical force to the brain resulting in at least one clinical sign (such as LOC, altered mental status, amnesia, or neurological signs) and two or more acute symptoms (e.g., headache, confusion, dizziness, or emotional changes)^6^. It is classified as “mild” unless LOC exceeds 30 min, post-traumatic amnesia lasts over 24 h, or the Glasgow Coma Scale score falls below 13 more than 30 min after injury^6^. This review was registered on PROSPERO, which can be accessed at https://www.crd.york.ac.uk/PROSPERO/view/CRD420250655304.

### Search strategy

The review was conducted in accordance with PRISMA (Preferred Reporting Items for Systematic Reviews and Meta-Analyses) guidelines. A comprehensive search was performed in the PubMed/MEDLINE and Embase databases for studies published between January 1990 and December 2024. A librarian was consulted to optimize the search strategy. Articles identified through the initial search were screened for inclusion by two independent reviewers (J.O. and V.J.). Any disagreements regarding the inclusion of full-text articles were reached with the assistance of senior reviewers.

Assessment of conflict of interest, funding for study and study design were assessed according to QUADAS criteria shown in Table S1 in the supplementary information.

### Study selection

The search strategy included the key terms: traumatic brain injury, concussion, loss of consciousness, outcome measures, and clinical outcomes. Variations of these terms were applied across the databases as appropriate. Additionally, an ancestor search was conducted by examining the references of the retrieved articles to identify any relevant studies that may have been missed in the original search.

**Fig. 1:**
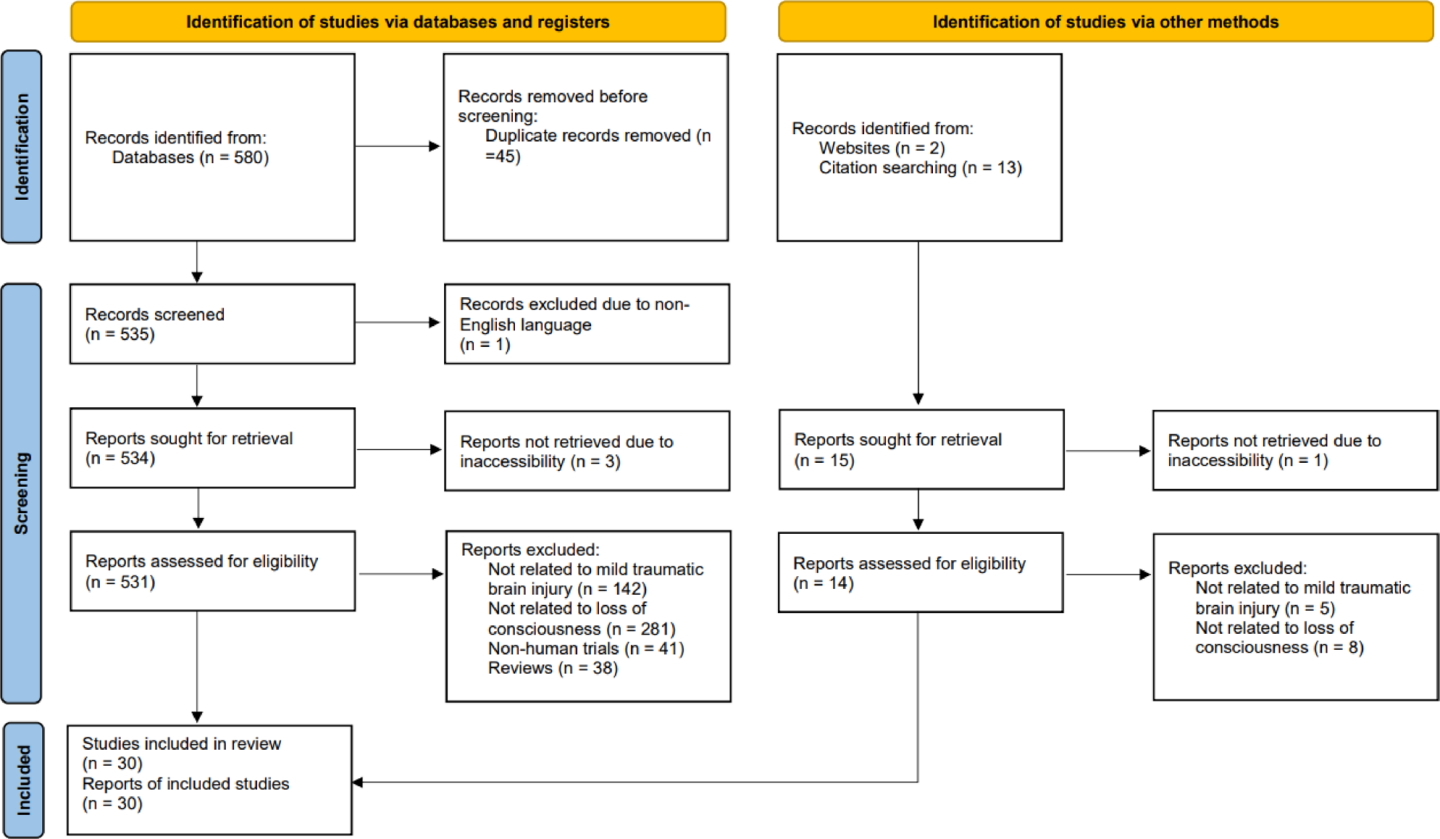
Preferred reporting items for systematic reviews and meta-analyses flowchart.

The exclusion criteria included all publications not related specifically to mTBI or LOC. Duplicate articles, non-human studies, review studies and meta-analyses, publications not in English, and studies without full text were also excluded. Figure 1 provides the PRISMA flow diagram.

### Data analysis

To assess whether loss of consciousness (LOC) in mTBI predicts clinical outcomes, we performed separate meta-analyses for each outcome category, comparing mTBI cases with and without LOC. For each included study, odds ratios (ORs) and their corresponding 95% confidence intervals (CIs) were extracted for both groups. Log-transformed ORs and variances were calculated to normalize the data for meta-analytic pooling.

Given the variability expected among studies, we applied a random-effects model using the DerSimonian and Laird method, which accounts for both within-study and between-study heterogeneity^11^. Pooled ORs and 95% CIs were obtained by exponentiating the combined log-transformed values, providing a comparative measure between LOC and non-LOC groups.

Heterogeneity was evaluated using the I² statistic to quantify the proportion of variance attributable to heterogeneity, and Cochran’s Q test to assess its significance, with p-values < 0.05 indicating substantial heterogeneity. In cases where a meta-analysis was not possible due to high heterogeneity or insufficient data, narrative synthesis was conducted to describe the study findings qualitatively.

## Results

Among 595 publications, a total of 30 studies were included in the final analysis and can be found in Table S2 in the supplementary information. The combined sample size for those studies was 527,004 individuals with history of mTBI. Military personnel or veterans comprised the investigated population in eleven studies^12–22^ and an elderly cohort comprised five studies^22–26^. Eight studies investigated the general population^9,27–34^ three covered a pediatric cohort^35–37^ and three looked at adult and college athletes^38–40^. Study-level forest plots by clinical outcome following mTBI with LOC are provided in Fig. S1 in the supplementary information.

### Persisting symptoms after concussion

Persisting symptoms after concussion (i.e., headache, dizziness and fatigue) were specifically covered in six studies^9,13–15,27,38^. Meta-analysis results indicate that LOC in mTBI significantly increases the likelihood of PSaC, with a pooled OR of 1.89 (95% CI: 1.59–2.25), and no significant heterogeneity across studies (*I²* =0%; Cochran’s Q = 2.73, *P* > 0.05). Only one study found no correlation between LOC and PSaC after mTBI^38^.

### Somatic and cognitive symptoms

Beyond PSaC studies have extensively examined somatic and cognitive symptoms after mTBI with LOC. Four studies showed that individuals with LOC after mTBI were three times more likely to experience somatic symptoms, including nausea, dizziness, headache, tinnitus or pain, than those without LOC (pooled OR 3.09, 95% CI: 1.94–4.96; moderate heterogeneity, *I²* =28.2%; Cochran’s Q = 3.14, *P* > 0.05)^16,17,28,37^. Similarly, cognitive symptoms, including attention and concentration difficulties, were also significantly associated with LOC, as demonstrated by a pooled OR 2.30 (95% CI: 1.65–3.19), with heterogeneity statistics showing (*I²* =31%; Cochran’s Q = 1.87, *P* > 0.05) indicating a consistent effect across studies^16,18,29,37^. Memory, a key cognitive domain, was implicated in three studies, with individuals with LOC being over twice as likely to experience short term memory deficits during acute or subacute stage of mTBI compared to those without LOC (pooled OR of 2.40 (95% CI: 1.72–3.35); heterogeneity analysis revealed (*I²* =24.5%; Cochran’s Q = 0.941, *P* = 0.62), demonstrating consistency across studies^16,19,27^.

### Recovery

The presence of LOC was strongly associated with prolonged symptom persistence and recovery, with patients experiencing significantly longer recovery times (Hazard ratio of 0.54)^36^. Athletes with LOC following mTBI experienced delayed neurological recovery, with a median symptom duration of 10 days vs. 5 days for those without LOC, and were 4.15 times more likely to have prolonged recovery (OR = 4.15, 95% CI: 2.12–8.15, *P* < 0.0001)^40^.

### Post-traumatic stress disorder

Seven studies examined an association between mTBI with LOC and PTSD^14,16–18,20,29,30^. The pooled analysis showed that individuals with LOC had 81% higher odds of developing PTSD compared to those without LOC (OR = 1.81, 95% CI: 1.54–2.12), with moderate heterogeneity across studies *I²* =45%; Cochran’s Q = 9.64, *P* = 0.7.

### Depression and anxiety

Depression was evaluated in five studies with a combined sample size of 1,336 participants, showing that LOC after mTBI was associated with 2.69 times higher odds of depression compared to those without LOC (pooled OR = 2.69; 95% CI: 2.10–3.43)^16,17,20,29,31^. Heterogeneity analysis showed no significant variability across studies (*I*² =0%, Cochran’s Q = 3.37, *P* > 0.05), indicating consistency in the association between LOC and depression. Furthermore, individuals with history of LOC had a higher likelihood of anxiety compared to those without LOC. In the Vanier study, the odds of anxiety were 1.50 times higher (95% CI: 1.00–2.23), while in the Dams-O’Connor study, individuals with a history of LOC were 2.59 times more likely to experience anxiety^29,31^. Patients with LOC also scored significantly higher on fear avoidance behavior, with an increase of about 1.7 points on the FAB-TBI scale (*P* = 0.002), suggesting a link between LOC and heightened symptom concerns or caution about activity exacerbation post-mTBI compared to those without LOC^32^.

### Health-related quality of life

Health-related quality of life (HRQoL) outcomes pertaining to either physical or mental health were examined in six studies^9,16,20,21,31,37^. All studies found consistently worse HRQoL outcomes among individuals with a previous mTBI with LOC. The meta-analysis of pooled results showed that individuals with LOC had 1.84 times higher odds of adverse outcomes compared to those without LOC (95% CI: 1.49–2.26). Moderate heterogeneity was observed across studies (*I²* =37.6%, Cochran’s Q = 5.05, *P* > 0.17), indicating that the variability in effect sizes was consistent with random sampling error and the results are reasonably consistent.

### Dementia and biomarkers of neurodegeneration

**Fig. 2:**
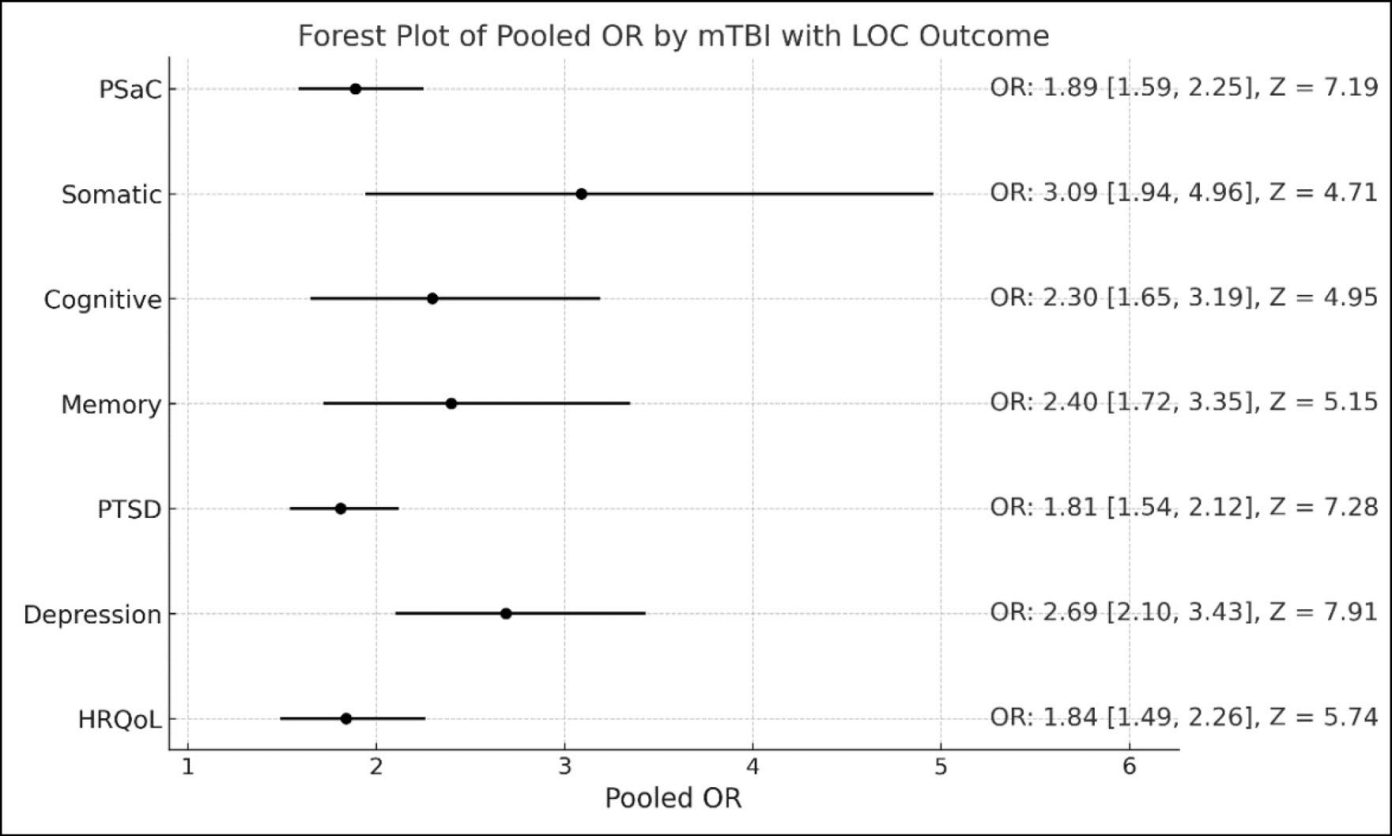
Forest plot of pooled OR by mTBI with LOC outcome.

Dementia and Alzheimer’s disease-related characteristics were examined in five studies^22–25,33^. In a study of 23,233 veterans, a history of mTBI with LOC significantly increased the risk of dementia (HR 2.51, 95% CI: 2.29–2.76) compared to those without LOC^22^. Yang et al. found that 83.3% of dementia cases had experienced mTBI with LOC, which was associated with a 12.5-fold increased risk of dementia (OR 12.5, 95% CI: 1.20–130.62, *P* = 0.035)^25^. Similarly, participants with TBI and LOC in the Agrawal et al. study had a greater amyloid-β load at autopsy (estimate = 0.25, 95% CI: 0.06–0.43, *p* = 0.008), further supporting the association between LOC and dementia-related pathology^23^. In a study of 7,130 elderly participants, LOC was associated with an increased risk of Lewy bodies in the frontal or temporal cortex (RR 1.59, 95% CI: 1.06–2.39, *P* = 0.025) compared to those without LOC in mild head injury^24^. One study found no association between LOC in mTBI and dementia^33^. Figure 2 provides forest plot of pooled OR by mTBI with LOC outcome.

Neuropathological changes and neurobiological disruptions associated with LOC were assessed across six studies, revealing significant findings about biomarkers, structural brain changes, and clinical outcomes. O’Brien et al. found that levels of GFAP and NfL were significantly higher in participants with LOC, indicating prolonged astroglial and axonal injury^39^. GFAP remained elevated at 4 weeks (mean difference: 0.34 pg/mL, 95% CI: 0.13–0.55), and NfL was elevated through 12 weeks (mean difference: 0.54 pg/mL, 95% CI: 0.22–0.85), supporting the need for extended recovery periods and cautious return-to-play decisions^39^. Structural brain changes in participants with LOC included gray matter reductions from 6 weeks to 12 months post-injury, particularly in the Rolandic operculum (23.04%) and inferior frontal gyrus (21.52%), along with white matter decreases in the superior longitudinal fasciculus (33.39%) and arcuate fasciculus (18.83%)^28^. In veterans, long-term changes after mTBI with LOC were linked to executive function impairment and white matter degradation, including higher radial diffusivity in the ventral prefrontal white matter (0.57 × 10⁻³ mm²/s, *P* = 0.02) and lower fractional anisotropy in the corpus callosum (genu: 0.64 vs. 0.67, *P* = 0.03) and posterior cingulate cortex (0.39 vs. 0.42, *P* = 0.03)^26^.

Franke et al. found that LOC after mTBI was associated with widespread reduced beta power, particularly in central and posterior regions, persisting over a decade post-injury, indicating long-term disruptions in attention and sensory processing^12^. Additionally, chronic alpha–beta spectral shifts reflected ongoing impairments in cognitive control and attention regulation^12^.

### Seizures

Rummala et al. found that mTBI with LOC increased the likelihood of posttraumatic seizures 1.4 times compared to those injuries without LOC (OR 1.4, 95% CI: 1.3–1.5, *P* < 0.001), with higher risk in children aged 0–5 years (OR 2.0, 95% CI: 1.9–2.1, *P* < 0.001) and those with three or more comorbidities, such as epilepsy or cerebral palsy (OR 4.0, 95% CI: 3.6–4.5, *P* < 0.001)^35^.

### Intracranial hemorrhage and infarction

In elderly patients, Turcato et al. found that those with LOC who were on direct oral anticoagulants had a 2.63 times higher likelihood of developing intracranial hemorrhage compared to those without LOC (OR 2.63, 95% CI: 1.04–6.62, *P* = 0.04)^34^. Finally, Agrawal et al. found that individuals with TBI and LOC had a 1.45 times higher likelihood of gross infarcts (OR 1.45, 95% CI: 1.04–2.02, *P* = 0.02) and a 1.70 times higher likelihood of microinfarcts (OR 1.70, 95% CI: 1.21–2.38, *P* = 0.002), particularly subcortical microinfarcts (OR 1.85, 95% CI: 1.23–2.79, *P* = 0.002) compared to those without LOC^23^.

## Discussion

Approximately 15% of patients diagnosed with mTBI experience persistent and disabling symptoms, including reduced functional ability, heightened emotional distress, and delayed return to work or school three months or more after injury^41^. In the present meta-analysis, PSaC and resulting somatic and cognitive difficulties were evaluated in half of the studies identified. To a lesser extent, PTSD, depression and related neuropsychological outcomes were reported in 24% of studies and longer-term neuropathological outcomes of dementia and PTSD were also reported in 26% of studies, demonstrating the role of LOC in worsening injury outcomes and resulting in delayed return to work.

### Persisting symptoms after concussion

As many as 40–50% of all patients with mTBI develop PSaC for up to three months, with 25% struggling with symptoms one year after the event^42^. Symptoms span physical, cognitive, and emotional domains^43^ and can result in significant occupational dysfunction, with 17% unable to work a year post-injury^3^. The most commonly reported symptoms include headaches, dizziness, and fatigue, along with memory and concentration difficulties, irritability, anxiety, and sleep disturbances^9,14,15^.

The presence of LOC has been strongly associated with prolonged recovery and higher severity of PSaC. Individuals with a history of mTBI and LOC are 70% less likely to return to work by six months post-injury^31^. Among military populations, LOC doubled the risk of PSaC^14^ and increased symptom severity after mTBI, particularly following blast-related injuries^13,15^. In civilian studies, LOC was present in approximately 20% of mTBI cases and doubled the odds of PSaC compared to cases without LOC^9^. Of the demographic predictors, female sex and a history of mTBI were significantly associated with increased risk of PSaC, with odds ratios of 1.46 and 1.59, respectively^13^.

Pediatric studies further highlight the importance of LOC, revealing that children aged 8–15 years with mTBI and LOC are nearly six times more likely to require additional educational interventions three months post-injury (OR: 5.94, 95% CI: 1.54–22.86)^37^. LOC should be a critical consideration in predicting recovery trajectories and guiding management decisions for mTBI.

### Post-traumatic stress disorder

PTSD affects nearly one-third of United States military veterans, significantly impacting quality of life and healthcare costs^44^. Veterans with PTSD face a doubled risk of unemployment and are nearly four times as likely to have a disability^45^. Among civilians, up to 40% of individuals with PTSD following mTBI have not returned to work by six months post-injury^12^. The economic burden of PTSD in the United States was estimated at $232.2 billion in 2018, including costs of $25,684 per military member and $18,640 per civilian with PTSD^46^. PTSD symptoms include intrusive thoughts, nightmares, avoidance behaviors, negative mood alterations (such as depression and emotional numbness), hyperarousal (including irritability and hypervigilance), and sleep disturbances, each contributing to long-term disability and functional impairment^14,18,30^. Notably, females with repetitive mTBI were twice as likely to exceed clinical thresholds for post-concussive, anxiety, and PTSD symptoms compared to males with repetitive mTBI or females with a single mTBI^47^. LOC has been identified as a significant marker of prolonged recovery and PTSD severity following head injury. In a study of 1,656 service members, those with LOC were twice as likely to be discharged due to disability compared to those with mTBI without LOC, with extracranial injury severity contributing to worse outcomes^14^.

Depression and neuropsychiatric symptoms are quite common after mTBI with a prevalence of 15.3% and constitute risk factors for poor recovery^48^. Risk factors for developing major depression in mTBI within 3 months of injury include older age and abnormal computerized tomography scans, with an OR of 7.8 for the latter^49^. Injury with LOC was linked to an almost 4-fold increase (OR 3.67) in diagnosis of depression^16^. Specifically, those with a previous head injury with LOC were more likely to report a history of anxiety disorder, depression, sleep disorder, and bipolar disorder and were slightly more likely to have a history of schizophrenia^31^. These findings align with similar studies reinforcing the notion that that mTBI with LOC shouldn’t be viewed as an acute event but rather as a long-term condition^50,51^.

### Health-related quality of life

TBI remains the leading cause of long-term impairments and disability in functional, physical, emotional, cognitive, and social domains, resulting in the annual cost of more than 221 billion United States dollars for disability, missed days at work, and lack of productivity^52^. In one study, nearly 60% of patients with mTBI cannot return to work two weeks post-injury, while almost 20% are unable one year after injury and report a congruent income decline^53^. HRQoL measures are negatively affected after mTBI including both physical (vision, balance, and pain) and mental health (depression, irritability, and apathy) domains^54^. Physical recovery from mTBI typically proceeds most rapidly in the first 3–6 months post-injury, yet psychosocial difficulties have shown less recovery at six months than physical problems^55^.

Injuries associated with LOC carry a greater risk of physical HRQoL decrements compared to injuries without LOC^21^. The presence of LOC in mTBI tripled the risk of depression and was a significant predictor of prolonged symptoms and lower HRQoL than those without LOC^9,21^. In addition, patients with previous TBI with LOC experienced worse outcomes at six months, including cognitive, mood, and life satisfaction domains, and were less likely to return to work at the 6-month follow-up (OR of 0.30) than those without a history of TBI with LOC^31^. Decreased HRQoL status, including emotional symptoms such as irritability, sadness, nervousness, or being ‘more emotional’, has been most predictive of delayed return to work^56^. Particular attention on the role of LOC may be further studied in female patients with mTBI.

### Dementia and neuropathological outcomes

Mounting epidemiological evidence suggest that moderate or severe TBI is an important risk factor for neurodegenerative diseases, including dementia, chronic traumatic encephalopathy, and even Parkinson’s disease^22,57,58^. The present review supports the finding that presence of LOC in mTBI also increases susceptibility to late-life neurodegenerative and vascular brain pathologic outcomes associated with dementia^22–25^.

Dementia has also been shown to be diagnosed 1.5 years earlier in those with mTBI compared to individuals without mTBI^22^. A study of neuropathological changes in 1,689 individuals found that mTBI with LOC was associated with greater cerebral amyloid-beta load and evidence of one or more gross and micro-infarctions compared to those without LOC^23^. In addition to vascular brain changes, white matter alterations and gray matter atrophy have been reported following mTBI with LOC, with cortical myelin reorganization occurring up to one year post-injury^28^. Long-term effects of repeated mTBI were further evidenced by alterations in electroencephalographic activity. Specifically, individuals with mTBI and LOC demonstrated higher alpha power and reduced beta power, indicative of decreased cognitive attention and focus^12^.

### Limitations

Several limitations of this meta-analysis warrant consideration. First, the meta-analyses were limited by the heterogeneity of the included studies, particularly in terms of participant characteristics (e.g., age, comorbidities, and injury mechanisms), outcome definitions, and follow-up durations. Although a random-effects model was applied to account for between-study variability, residual heterogeneity may still influence the pooled estimates. Second, while we aimed to include all relevant studies, the selection process may have introduced biases. For instance, only studies published in English were included, potentially excluding relevant research in other languages, although this limitation has previously been shown to have minimal impact on findings^59^.

## Conclusion

mTBI with LOC is associated with an increased risk of adverse prolonged clinical outcomes when compared to mTBI without LOC. Both the presence and duration of LOC appear to signify a more severe form of injury, with implications for higher rate of post-TBI sequelae including concussive symptoms, PTSD, depression, and neurodegenerative disease risk, that affect HRQoL. Given the heightened risk of deleterious outcomes, clinical assessments should consider incorporating the presence and duration of LOC to designate those patients at higher risk of post-TBI sequelae, adjudicate return to play, and consider supportive therapy.

## Supplementary Information

Below is the link to the electronic supplementary material.

Supplementary Material 1

## Data Availability

The datasets used and/or analyzed during the current study are available from the corresponding author on reasonable request.

## Abbreviations

CI: Confidence interval
HR: Hazard ratio
HRQoL: Health related quality of life
LOC: Loss of consciousness
mTBI: Mild traumatic brain injury
OR: Odds ratio
PRISMA: Preferred reporting items for systematic reviews and meta-analyses
PSaC: Persisting symptoms after concussion
PTSD: Posttraumatic stress disorder
TBI: Traumatic brain injury
