# Supplementary material for: Systematic review and meta-analysis of the impact of loss of consciousness on clinical outcomes in mild traumatic brain injury
Source: Sci Rep. 2025 Aug 12;15:29531. doi: 10.1038/s41598-025-13979-0 (PMC12343850; doi:10.1038/s41598-025-13979-0)
Supplement: Supplementary file 1 — Supplementary Material 1 [file 41598_2025_13979_MOESM1_ESM.pdf]

Supplementary information

Table S1. QUADAS Criteria for All Studies

QUADAS Question	Yes	No	Unclear
Was the spectrum of patients' representative of the patients who will receive the test in practice?	30		
Were selection criteria clearly described?	27	3	
Is the reference standard likely to correctly classify the target condition?	30		
Is the time period between reference standard and index test short enough to be reasonably sure that the target condition did not change between the two tests?	28		2
Did the whole sample or a random selection of the sample, receive verification using a reference standard of diagnosis?	30		
Did patients receive the same reference standard regardless of the index test result?			30
Was the reference standard independent of the index test (i.e. the index test did not form part of the reference standard)?	30		
Was the execution of the index test described in sufficient detail to permit replication of the test?	27		3
Was the execution of the reference standard described in sufficient detail to permit its replication?	27		3
Were the index test results interpreted without knowledge of the results of the reference standard?			30
Were the reference standard results interpreted without knowledge of the results of the index test?			30
Were the same clinical data available when test results were interpreted as would be available when the test is used in practice?	30		
Were uninterpretable/intermediate test results reported?	16		14
Were withdrawals from the study explained?	30		

Table S2. Summary of studies of mTBI and LOC

Author, year	Study type	Sample size, age	Grading Scale	Primary Outcome	Mechanism	Follow Up	Key findings	Level of evidence
Ponsford et al., 2019[9]	Pro	n=343, 18-99	GCS, LOC	PSaC, HRQoL	PSaC Questionnaire	7 months	LOC and pre-injury psychological issues are associated with persistent symptom reporting	Level II
Franke et al., 2023[12]	Pro	n=340, 18+	GCS, LOC, PTA, CT	Executive functioning	EEG	11 years	PTA + LOC resulted in poorer cognitive performance and reduced power in beta frequencies.	Level II
Stein et al., 2016[13]	Pro	n = 4518, N/A	LOC, lapse of memory	PSaC	Questionnaire	1, 3, 9 months	MTBI triples the risk of PSaC. LOC or lapse of	Level II

							memory increased the severity of PSaC.	
Eskridge et al., 2013[14]	Retro	n=1656, 22-25	GCS, AIS	Mental health	Data from medical files	N/A	LOC and altered mental state were significant predictors of PTSD and PSaC	Level III
Wilk et al., 2010[15]	Pro	n=3952, N/A	LOC,	PSaC, PTSD	Anonymous survey	N/A	Blast mechanism of concussion was inconsistently associated with PSaC	Level II
Hoge et al., 2008[16]	Pro	n=2714, ≤30	GCS, LOC	PSaC	Anonymous survey	N/A	Soldiers with mTBI with LOC reported poor general health, missed work days, number of somatic and PSaC.	Level II
Kim et al.,2023[17]	Retro	n = 157, avg 28	LOC	PTSD, depression	Self-reported questionnaire	3 & 12 months	PTSD rates elevated in the mTBI with LOC group (OR3.87). Depression higher in mTBI with LOC (OR 4.83)	Level III
Waters et al., 2024[18]	Pro	n=77,<br><br>avg 32	LOC	PTSD	Questionnaire	N/A	White matter variability associated with LoC in mTBI is associated with increased PTSD.	Level II
Norris et al., 2014[19]	Retro	n=210, avg 24	LOC	PSaC, memory	Data from medical files	N/A	LOC impacts cognitive dysfunction	Level III
Vasterling et al., 2018[20]	Pro	n=760, N/A	N/A	PSaC	Self- surveys	N/A	MTBI of deployed service members typically have limited neuropsychological consequences.	Level II
MacGregor et al., 2023[21]	Pro	n = 810, avg. 27.2	LOC	HRQoL, PTSD, PSaC	36-Item Short Form Health Survey	5.8–11.5 years	TBI with LOC was significantly associated with lower HRQoL. Symptoms of PTSD and depression were the strongest predictors of lower HRQoL.	Level II
Barnes et al., 2018[22]	Retro	n = 357,558, avg 49.5	GCS, LOC	Dementia	International Classification of Diseases	4.2 years	Adjusted hazard ratios for dementia were 2.36 for mTBI without LOC, 2.51 for mTBI with LOC, 3.19 for mild TBI with LOC status unknown.	Level III
Agrawal et al., 2022[23]	Retro	n = 1689, avg at death 89.2	LOC	Neuro-pathologic measures	Self-reported questionnaire	8 years	Patients with TBI with LOC had a greater amyloid-β load and higher odds of having gross infarcts and microinfarcts, particularly subcortical microinfarcts.	Level III

Crane et al., 2016[24]	Pro	n = 7130, avg 79.9	LOC	Dementia, AD	Clinical data	4-11 years	TBI with LOC increases risk for Lewy bodes, PD, but not dementia, AD, neuritic plaques, or neurofibrillary tangles.	Level II
Yang et al., 2015[25]	Pro	n=27, avg 60	LOC	Dementia	Amyloid PET imaging.	N/A	LOC as a significant risk factor for developing dementia post-mTBI.	Level II
Sorg et al., 2014[26]	Pro	n=30, avg 30.7	AOC, LOC, GCS, PTA	Executive functioning	Brain imaging	N/A	History of mTBI and LOC may be a risk factor for reduced EF as well changes to ventral prefrontal WM	Level II
Roy et al.,2019[27]	Pro	n=407, avg 43	LOC, GCS	PSaC, depression	Questionnaire	1 & 6 months	LOC was strongly associated with PSaC	Level II
Muller et al., 2021[28]	Pro	n = 85, avg 34.5	LOC, GCS	The brain's GM and WM	Voxel-based-morphometry	1 – 12 months	Patients with uncomplicated mTBI, showed significant GM decreases. MTBI patients experienced significant WM.	Level II
Vanier et al., 2024[29]	Retro	n=524, avg 42	LOC	PTSD, depression	Data from medical files	N/A	LOC in MTBI doubles the risk of somatic or cognitive outcomes.	Level III
Roitman et al., 2013[30]	Retro	n = 287, avg 36	LOC	PTSD	Posttraumatic Stress Symptom Inventory Interviews	10 days 8 months	LOC doubles the risk of PTSD compared to non-LOC injuries	Level III
Dams-O'Connor et al., 2013[31]	Pro	n=586, 16+	GCS, LOC, ISS	PSAC	Telephone survey	6 months	Previous TBI may lead to worse PSaC outcomes even after mTBI	Level II
Caircross et al., 2021[32]	Retro	n=563, avg 39	LOC	Fear Avoidance Behavior	Questionnaire	N/A	Patients with LOC had significantly higher fear avoidance behavior post-mTBI	Level III
Mehta et al., 1999[33]	Pro	n=797 55+	LOC, SRT	PSaC, Dementia, AD	Questionnaire	1,3,5 years	LOC in mTBI is not predictive of dementia or AD.	Level II
Turcato et al., 2015[34]	Retro	n=930, avg 83	LOC	ICH	CT scans	24 hrs	LOC associated with higher prevalence of ICH	Level III
Rumalla et al., 2018[35]	Retro	n = 124,444, < 21 years	Hours and baseline return	PSaC	Kids' Inpatient Database	N/A	Risk factors for PTS included age 0–5 years, preexisting comorbidities, shaken infant syndrome, subdural hematoma, closed-type injury, and LOC.	Level III

Fehr et al., 2019[36]	Retro	n=431, age 18.	LOC, PTA	PSaC	Questionnaire	N/A	Prolonged mTBI symptoms is related to symptom severity, female sex and LOC	Level III
Yeates et al., 2012[37]	Pro	n=285, ave. 15	LOC, GCS	HRQoL	Parents rated injuries	1 year	Children with mTBI show increase in functional impairment	Level II
Teel et al., 2017[38]	Pro	n=8905, 18 +	LOC, PTA	HRQoL	Questionnaire	90 days	Amnesia resulted in slower recovery, cognition, and balance. Previous concussions had poorer balance than those with no previous history.	Level II
O'Brien et al.,2023[39]	Pro	n=8,<br><br>avg 23	LOC	Neuro-pathologic measures	Questionnaire	6 months	LOC and extended<br><br>biomarker elevations support the use of LOC for informing return-to-play timelines.	Level II
McCrea et al., 2013[40]	Pro	n=570, avg 19	LOC	PSaC	Graded Symptom Checklist	90 days	LOC had over 4 times the risk of prolonged recovery compared to those without LOC.	Level III

Note: Pro (prospective); Retro (retrospective); LOC (loss of consciousness); GSC (Glasgow Coma Scale); PSaC (Persisting Symptoms after Concussion); AD (Alzheimer's disease); PTSD (Post-traumatic Stress Disorder), PTA (Post Traumatic Amnesia); ICL (Intracerebral lesion); GM (grey matter); WM (white matter); OR (odds ratio), HRQoL (Health-Related Quality of Life); ICH (Intracranial Hemorrhage); WCST (Wisconsin Card Sorting Test), COWAT (Controlled Oral Word Association Test), STAI (State-Trait Anxiety Inventory), SRT (Selective Reminding Test) and EEG (electroencephalogram).

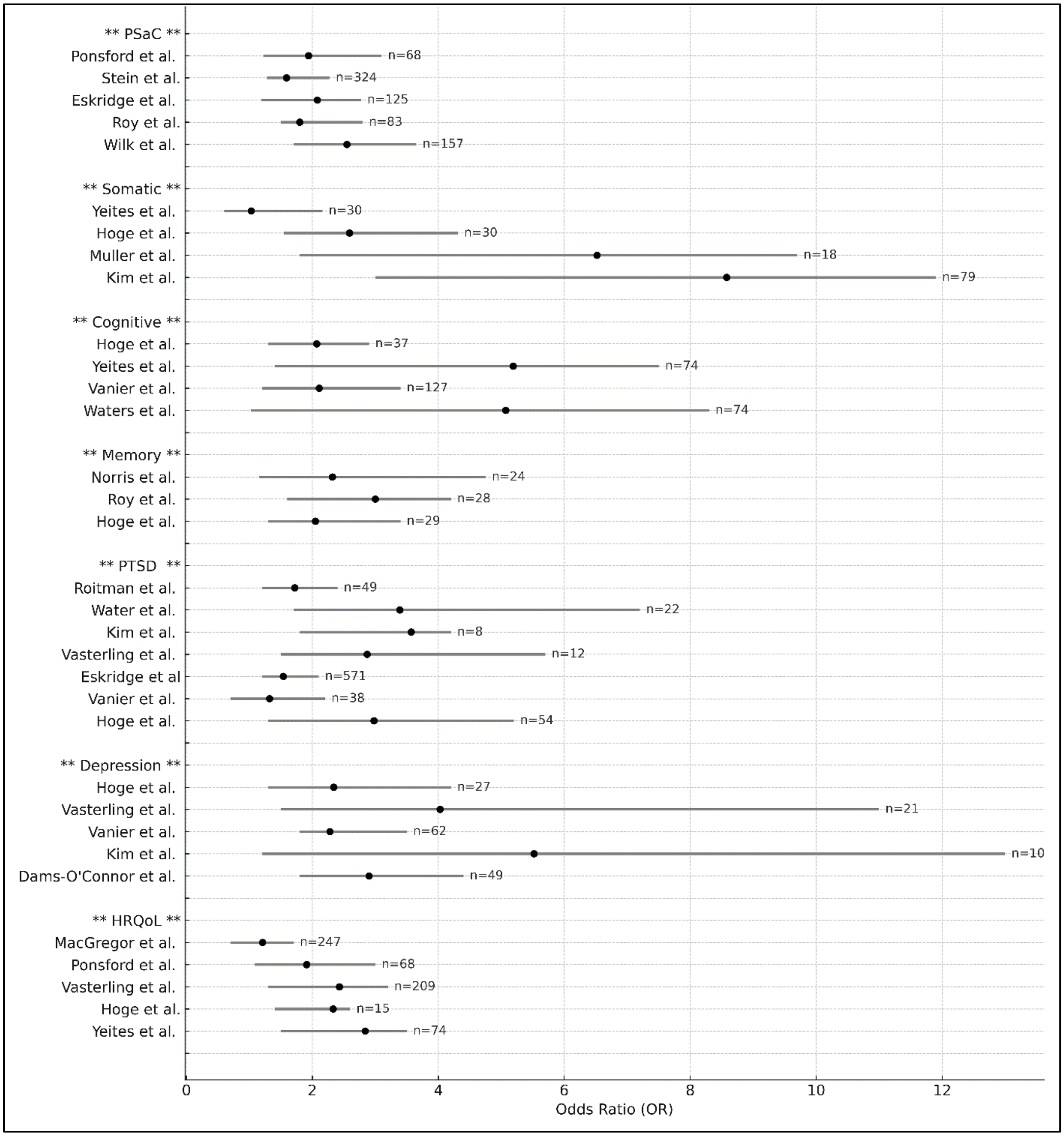

Figure S1: Study-level forest plots by clinical outcome following mTBI with LOC
